# Self-reported sleep duration mitigates the association between inflammation and cognitive functioning in hospitalized older men

**DOI:** 10.3389/fpsyg.2015.01004

**Published:** 2015-07-21

**Authors:** Joseph M. Dzierzewski, Yeonsu Song, Constance H. Fung, Juan C. Rodriguez, Stella Jouldjian, Cathy A. Alessi, Elizabeth C. Breen, Michael R. Irwin, Jennifer L. Martin

**Affiliations:** ^1^Geriatric Research, Education and Clinical Center, VA Greater Los Angeles Healthcare SystemLos Angeles, CA, USA; ^2^David Geffen School of Medicine, University of California, Los AngelesLos Angeles, CA, USA; ^3^Cousins Center for Psychoneuroimmunology, Semel Institute for Neuroscience and Human Behavior, University of California, Los AngelesLos Angeles, CA, USA

**Keywords:** sleep duration, inflammation, cognition, hospitalization, older adults

## Abstract

Examination of predictors of late-life cognitive functioning is particularly salient in at-risk older adults, such as those who have been recently hospitalized. Sleep and inflammation are independently related to late-life cognitive functioning. The potential role of sleep as a moderator of the relationship between inflammation and global cognitive functioning has not been adequately addressed. We examined the relationship between self-reported sleep duration, inflammatory markers, and general cognitive functioning in hospitalized older men. Older men (*n* = 135; Mean age = 72.9 ± 9.7 years) were recruited from inpatient rehabilitation units at a VA Medical Center to participate in a cross-sectional study of sleep. Participants completed the Mini-Mental State Examination and Pittsburgh Sleep Quality Index, and underwent an 8 a.m. blood draw to measure inflammatory markers [i.e., C-reactive protein (CRP), tumor necrosis factor alpha (TNFα), soluble intercellular adhesion molecule-1 (sICAM-1), and interleukin-6 (IL-6)]. Hierarchical regression analyses (controlling for age, education, race, depression, pain, health comorbidity, and BMI) revealed that higher levels of CRP and sICAM are associated with higher global cognitive functioning in older men with sleep duration ≥6 h (β = −0.19, β = −0.18, *p*'s < 0.05, respectively), but not in those with short sleep durations (*p*'s > 0.05). In elderly hospitalized men, sleep duration moderates the association between inflammation and cognitive functioning. These findings have implications for the clinical care of older men within medical settings.

## Introduction

Cross-sectional and longitudinal studies demonstrate a decline in cognitive functioning with advanced age. Identification of predictors of cognitive functioning in older adults is a key step in efforts to prolong late-life vitality. Both sleep (Blackwell et al., 2006; McCrae et al., 2012) and inflammation (Weaver et al., 2002; Teunissen et al., 2003; Baune et al., 2008) have demonstrated inconsistent associations with late-life cognitive functioning. We sought to clarify the relationships among sleep, inflammation, and cognitive functioning in older adults for whom impairments in cognitive functioning are particularly salient, in this case hospitalized patients (Wilson et al., 2012).

Advanced age is associated with changes in sleep duration, quantity, and architecture (Morgan, 2000). These age-related changes in sleep appear to be magnified in inpatient settings, where older adults commonly suffer from extremely fragmented sleep and have short nighttime sleep duration (Drouot et al., 2008; Isaia et al., 2011). While disturbed sleep in these settings predicts increased mortality risk within 1 year (Martin et al., 2011), the relationship between sleep and cognitive functioning in the inpatient setting is unknown.

In community-dwelling older adults, habitual self-reported sleep duration has shown some promise as a predictor of global cognitive functioning. However, inconsistent results limit the conclusions that can be drawn regarding the relationship between sleep duration and cognitive functioning. For example, no association between sleep duration and executive functioning has been reported (Nebes et al., 2009), whereas, others have reported that older adults with short sleep durations have lower levels of global cognitive functioning (Tworoger et al., 2006; Kronholm et al., 2009). Still other researchers observed relationships between long sleep duration, but not short sleep duration, and general cognitive functioning (Faubel et al., 2009). Beyond our recent work demonstrating that when sleep improves following hospital discharge, so too does cognitive functioning (Dzierzewski et al., 2014), there is a dearth of research examining the relationship between sleep and cognitive functioning in hospitalized older adults. Such research is important, given the increased risk for negative cognitive changes associated with hospitalization in late-life (Wilson et al., 2012) and the relative ease of assessing self-reported sleep duration.

The relationship between markers of inflammation and cognitive performance is also not fully understood. Whereas, some longitudinal studies have indicated a relationship between high cytokine levels, for example, interleukin-6 (IL-6) and C-reactive protein (CRP), and development of multiple forms of dementia, data from the MacArthur Study of Successful Aging indicated that there was only a limited association between inflammatory factors and baseline cognitive function and the rate of longitudinal cognitive decline after correcting for potential confounders (Weaver et al., 2002; Teunissen et al., 2003; Baune et al., 2008). Furthermore, in older adults, higher levels of inflammatory markers have not been consistently associated with poorer cognitive functioning (Teunissen et al., 2003). In contrast to findings in healthy older adults, acute activation of the inflammatory cascade in the context of recent changes in health (e.g., infection, surgery) may be adaptive, serve to aid in recovery from sickness, and have a different relationship with cognitive functioning than chronic inflammation (Dantzer and Kelley, 2007; Terrando et al., 2011). As such, additional research is needed to determine the relationship between inflammation and late-life cognitive functioning in hospitalized older adults.

The inconsistent relationships independently reported between sleep and cognitive functioning and inflammation and cognitive functioning may be partially explained by a general lack of consideration for the relationships among sleep and inflammation. Sleep duration appears to be directly related to inflammation. Individuals with both irregularly short and long sleep durations have been found to exhibit heightened levels of inflammatory markers (Patel et al., 2009). As such, the relationship between inflammation and cognitive functioning may differ by sleep duration. As sleep in late-life is a relatively malleable behavior (Dzierzewski et al., 2010), examination of whether the relationship between inflammatory markers and global cognitive functioning differs by sleep duration is of theoretical and practical importance.

The current study aimed to examine the relationships among sleep duration, inflammation, and global cognitive function in older hospitalized men, accounting for the effects of age, education, race, medical comorbidities/health, depressive symptoms, pain, and body mass index (BMI). We hypothesized that shorter sleep duration would be associated with worse global cognitive functioning. In healthy, community-based samples of older adults it would be expected that higher levels of inflammatory markers [i.e., CRP, soluble intercellular adhesion molecule (sICAM), tumor necrosis factor-alpha (TNFα), and IL-6] would likely be related worse global cognitive functioning. In the unique context of late-life hospitalization, we hypothesized that there would be an association between levels of inflammatory markers and global cognitive functioning, but it was not clear in which direction this association might be expected to occur. Lastly, we examined whether the association between inflammation and global cognitive functioning differed by length of sleep duration. Given the prior research which revealed independent inconsistent associations between sleep duration and cognitive functioning and between inflammation and cognitive functioning, coupled with evidence that individuals with varying durations of sleep exhibit different inflammatory responses, we hypothesized that the relationship between inflammation and global cognitive functioning would differ in hospitalized older men with short sleep durations as compared to those with more normal sleep durations.

## Methods

### Participants

Participants included in the present analysis were drawn from two studies of sleep in older adults admitted for rehabilitation. The first study was a descriptive study of sleep and inflammatory markers (*n* = 36). The second study included the baseline phase of an intervention trial to improve sleep during post-acute rehabilitation (*n* = 151). Both studies were set in inpatient rehabilitation units at a Veterans Affairs Medical Center, and included 1 week of data collection. As such, they provide an opportunity to examine predictors of cognitive functioning in a unique sample of older adults who find themselves at an increased risk for poor cognitive outcomes. Inclusion criteria were identical for both studies, and included: (1) aged 60 years or older; and (2) admitted for rehabilitation (i.e., receiving physical or occupational therapy). Individuals were excluded if: (1) found to have profound cognitive impairment [defined by Mini-Mental State Examination (MMSE)<12]; (2) had resided in a nursing home prior to admission; (3) transferred, died, or were discharged within 1 week of admission; (4) could not participate in the study due to a severe medical illness or severe behavioral disturbance; and (5) were unable to communicate verbally in English during the screening process. Research methods were reviewed and approved by the Veterans Administration Greater Los Angeles Healthcare System Institutional Review Board (i.e., ethics committee), and informed consent was obtained from all participants.

### Procedures

Admission to the inpatient rehabilitation units at a Veterans Affairs Medical Center was continuously monitored during data collection to identify potential study participants. When a potential research participant was identified (based upon inclusion and exclusion criteria listed above), informed consent was obtained and the patient was formally enrolled in the study. After enrollment, all participants completed a comprehensive assessment including collection of demographic, clinical, sleep, and cognitive data, and measurement of inflammatory markers (details below). First, individuals completed the MMSE to establish eligibility and measure global cognitive functioning. Eligible participants then completed a series of questionnaires administered in interview format by a trained research assistant at the patient's bedside. On 1 day of monitoring, a blood sample was collected by a study phlebotomist at 8 a.m. to measure inflammatory markers.

### Measures

#### Global cognitive functioning

We administered the MMSE, a 20-item measure of general cognitive functioning; scores range from 0 to 30, with scores <24 suggestive of cognitive impairment (Folstein et al., 1975). MMSE total score was used as a measure of general/global cognitive functioning and to exclude those with profound cognitive impairment as described above.

#### Demographic data

Basic demographic information was recorded for all participants, including age, education (measured in reported years of formal education), and race (grouped as non-Hispanic white and all others).

#### Clinical data

Depressive symptoms were assessed with the 5-item version of the Geriatric Depression Scale (score range = 0–5; scores >2 suggest clinically meaningful depression) (Rinaldi et al., 2003). The experience of pain during the rehabilitation stay was assessed with a modified version of the Geriatric Pain Measure (GPM; higher scores indicate worse pain) (Ferrell et al., 2000). Illness severity and comorbidity burden during acute inpatient rehabilitation was computed with the Cumulative Illness Rating Scale for Geriatrics (CIRS-G) (Miller et al., 1992; Parmelee et al., 1995). This measure was completed by an experienced research registered nurse, using data gathered during a brief physical examination by a study physician and abstracted medical record data. The CIRS-G quantifies comorbidity through rating of 14 body systems (e.g., heart, respiratory, liver) on a continuum from no impairment to extremely severe impairment (Parmelee et al., 1995). BMI was calculated from measures of height and weight in patients' medical records.

#### Sleep

Self-reported sleep duration over the prior 7 days was assessed with the Pittsburgh Sleep Quality Index [PSQI; (Buysse et al., 1989)]. The PSQI asks “How many hours of *actual sleep* did you get at night?” Responses were categorized into the following mutually exclusive groups, according to PSQI scoring conventions (Buysse et al., 1989): (1) less than 5 h of sleep per night, (2) 5–6 h of sleep per night, (3) 6–7 h of sleep per night, and (4) more than 7 h of sleep per night. As such, the PSQI provides an index of sleep duration ranging from short sleep length to average sleep length.

#### Inflammatory markers

One blood sample was collected per participant into an anticoagulated (EDTA) tube at 8 a.m. on one morning of the study. Samples were centrifuged at 4°C, and aliquots of plasma were prepared for storage within 30 min of collection, and then immediately frozen at −70°C until assays were conducted. At the end of the studies, plasma samples were thawed and assayed for inflammatory markers. Each sample was assayed in duplicate using enzyme-linked immunosorbent assays (ELISAs), according to manufacturer's instructions except as noted. CRP levels were determined by a high sensitivity ELISA (Immundiagnostik, ALPCO Immunoassays, Salem, NH) at a routine sample dilution of either 1:200 or 1:500 (up to 1:2000 as needed), and with an extended standard curve, for an assay range of 0.2–300 mg/L (taking the sample dilution into account). Plasma levels of TNFα and IL-6 were determined by Quantikine High Sensitivity ELISAs (R&D Systems, Minneapolis, MN); IL-6 samples were routinely diluted 3-fold, and when necessary, further diluted up to 20-fold in order to measure samples with IL-6 concentrations up to 200 pg/mL (taking sample dilution into account). Measurement of sICAM-1 was performed using a regular sensitivity sICAM-1 ELISA (R&D Systems, Minneapolis, MN). For all four inflammatory markers, intra-assay variability was less than 6% and inter-assay variability was less than 8%.

### Statistical analyses

Data were analyzed with IBM SPSS 22 statistical software. Independent sample *t*-tests were used to compare characteristics of those with complete data (i.e., the included sample) and those with one or more missing data points (i.e., the excluded sample).

Four separate, 3-block hierarchical regressions were estimated to predict general/global cognitive functioning (i.e., MMSE total score). In Block 1, demographic (i.e., age, education, race) and clinical information (i.e., comorbidity/health, depression, pain, and BMI) were entered. In Block 2, sleep duration and a single inflammatory marker, either CRP (mg/L), sICAM-1 (ng/mL), IL-6 (pg/mL), or TNFα (pg/mL), were entered. In Block 3, the interaction term between sleep duration and the inflammatory marker (either CRP, sICAM-1, IL-6, or TNFα) was entered. The four separate hierarchical regression models only differed by the specific inflammatory marker included in the model. Models were evaluated based on indicators of model fit, while individual predictors were evaluated based on significance levels. Follow-up analysis involved examination of simple slopes and regions of significance (Preacher et al., 2006). To reduce the influence of extreme values, the highest 5% of inflammatory marker values (equivalent to 2 SDs) were replaced with the values at the 95th percentile (Field, 2005). All variables were transformed using the Blom transformation (Blom, 1958) prior to statistical analysis. For all analyses, *p* < 0.05 was considered statistically significant.

## Results

### Sample characteristics

In total, 187 older men were deemed eligible and provided at least some data. Of these, 135 (mean age 72.9 ± 9.7 years) provided complete data and were included in the present analysis. On average, these older men had above high school levels of education (mean education 13.9 ± 3 years), were predominantly non-Hispanic white (62%), had low levels of depressive symptoms (GDS-5 mean 1.5 ± 1.4), moderate levels of pain (mean GPM 50 ± 27.8), experienced moderate levels of physical comorbidities (mean CIRS-G 22 ± 5.4), and were overweight (mean BMI 27.0 ± 5.5). Median levels (and 25th–75th percentile ranges) of inflammatory markers were: CRP 19.0 (4.4–59.1) mg/L, sICAM-1 263 (221–337) ng/mL, IL-6 9.4 (5.3–16.6) pg/mL, and 1.9 (1.5–2.4) TNFα pg/mL. Distribution of sleep duration was as follows: 28% reported sleeping for more than 7 h per night, 20% reported sleeping between 6 and 7 h per night, 20% reported sleeping between 5 and 6 h per night, and 32% reported sleeping less than 5 h per night. As expected after excluding those with profound cognitive impairment, the sample was generally cognitively healthy (mean MMSE 25.8 ± 3.4). The only significant difference between individuals with incomplete data (*n* = 52) and complete data (*n* = 135) was that participants with incomplete data were older than those who provided complete data (*p* = 0.012, mean age = 76.5 years vs. 72.9 years, respectively). See Table 1 for complete listings of demographic and clinical data.

**Table 1:** **Patient demographics and descriptive variables**.

	**Included sample (*n* = 135)**		**Excluded sample (*n* = 52)**		***t/χ ^2^***	***df***	***P***
	**Mean**	**(SD)**	**Mean**	**(SD)**			
Age (years)	72.9	(9.7)	76.5	(8.8)	2.56	109.1^a^	0.01
Education (years)	13.9	(3.0)	13.1	(2.6)	−1.21	156	>0.05
Race (% White)	62.2%		61.5%		0.01	1	>0.05
GDS Score	1.5	(1.4)	1.4	(1.4)	−0.29	182	>0.05
GPM Score	50.0	(27.8)	46.1	(29.5)	−0.78	175	>0.05
CIRS Total Score	22.0	(5.4)	23.4	(5.6)	1.44	175	>0.05
BMI	27.0	(5.5)	28.2	(10.5)	0.74	50.88^a^	>0.05
Sleep Duration (% of respondents)					0.37	179	>0.05
Less than 5 h	32.9%		32.6%				
5 to 6 h	20.0%		21.7%				
6 to 7 h	20.0%		21.7%				
Greater than 7 h	28.1%		23.9%				
CRP [median (25%ile, 75%ile)]	19.0	(4.4, 59.1)	19.9	(5.8, 65.4)	0.22	183	>0.05
sICAM [median (25%ile, 75%ile)]	263	(221, 337)	247	(197, 330)	−1.39	183	>0.05
TNFα [median (25%ile, 75%ile)]	1.9	(1.5, 2.4)	1.9	(1.4, 2.6)	−0.46	183	>0.05
IL6 [median (25%ile, 75%ile)]	9.4	(5.3, 16.6)	10.7	(5.4, 20.5)	0.53	185	>0.05
MMSE Score	25.8	(3.4)	24.5	(4.3)	−1.93	76.62^a^	>0.05

*Race dichotomously coded as non-Hispanic white = 1, all other = 0; GDS, Geriatric Depression Scale, modified 5-item version; GPM, Geriatric Pain Measure; CIRS, Cumulative Illness Rating Scale; BMI, body mass index; CRP, C-reactive protein (mg/L); sICAM, soluble Intercellular Adhesion Molecule (ng/mL); TNFα, Tumor Necrosis Factor-alpha (pg/mL); IL6, Interleukin-6 (pg/mL)*.

a *degrees of freedom and t-statistic were adjusted for non-homogeneity of variance*.

### Hierarchical regressions predicting global cognitive functioning

Final model statistics are presented in Table 2. Age and racial group emerged as consistent predictors of MMSE scores across the different inflammatory marker regression models, suggesting that younger men within this study population, and non-Hispanic whites among all men, had higher (i.e., better) MMSE scores. Sleep duration was not a direct significant predictor of MMSE scores. Only sICAM was a significant independent predictor of MMSE scores, β = 0.21, *p* < 0.01, suggesting men with higher levels of sICAM during hospitalization had higher MMSE scores. The interactions between sleep duration and CRP, β = −0.19, *p* < 0.05, and sleep duration and sICAM, β = −0.18, *p* < 0 05 were significant, suggesting that the relationship between inflammation and global cognitive functioning differed by sleep duration groups. The CRP and sICAM models explained significant variance in global cognitive functioning, final model adjusted R^2^ of 21 and 22%, respectively. Likewise, the addition of the interaction term between sleep duration and the inflammatory marker resulted in significant improvement in model fit for both the CRP and sICAM models.

**Table 2:** **Summary of hierarchical regression analysis predicting general cognitive functioning (*n* = 135)**.

	**CRP**			**sICAM**			**TNFα**				**IL6**		
	**B**	**(SE)**	**β**	**B**	**(SE)**	**β**	**B**	**(SE)**		**β**	**B**	**(SE)**	**β**
**BLOCK 1**														
Age (years)	−0.29	(0.08)	−0.32^***^	−0.29	(0.08)	−0.32^***^	−0.29	(0.08)		−0.32^***^	−0.29	(0.08)	−0.32^***^
Education (years)	0.13	(0.08)	0.14	0.14	(0.08)	0.14	0.13	(0.08)		0.14	0.13	(0.08)	0.14
Race	0.26	(0.11)	0.19^*^	0.25	(0.11)	0.18^*^	0.26	(0.11)		0.19^*^	0.26	(0.11)	0.19^*^
GDS Score	−0.12	(0.09)	−0.11	−0.12	(0.09)	−0.11	−0.12	(0.09)		−0.11	−0.12	(0.09)	−0.11
GPM Score	−0.02	(0.08)	−0.02	−0.01	(0.08)	−0.01	−0.02	(0.08)		−0.02	−0.01	(0.08)	−0.01
CIRS Total Score	−0.12	(0.08)	−0.13	−0.13	(0.08)	−0.14	−0.12	(0.08)		−0.13	−0.13	(0.08)	−0.13
BMI	−0.02	(0.08)	−0.02	−0.02	(0.08)	−0.02	−0.02	(0.08)		−0.02	−0.02	(0.08)	−0.02
Adjusted R^2^	0.18			0.18			0.18				0.18		
F for Change in R^2^	5.05^***^			5.08^***^			5.05^***^				5.06^***^		
**BLOCK 2**														
Age (years)	−0.26	(0.09)	−0.28^**^	−0.27	(0.08)	−0.30^***^	−0.29	(0.08)		−0.32^***^	−0.29	(0.08)	−0.32^***^
Education (years)	0.14	(0.08)	0.15	0.12	(0.08)	0.13	0.13	(0.08)		0.14	0.14	(0.08)	0.15
Race	0.23	(0.11)	0.17^*^	0.20	(0.11)	0.14	0.26	(0.11)		0.19^*^	0.24	(0.11)	0.17^*^
GDS Score	−0.10	(0.09)	−0.09	−0.13	(0.09)	−0.13	−0.12	(0.09)		−0.12	−0.12	(.09)	−0.11
GPM Score	−0.02	(0.08)	−0.02	−0.01	(0.08)	−0.01	−0.01	(0.08)		−0.01	−0.01	(0.08)	−0.02
CIRS Total score	−0.14	(0.08)	−0.15	−0.15	(0.08)	−0.16	−0.12	(0.08)		−0.13	−0.14	(0.08)	−0.15
BMI	−0.02	(0.08)	−0.02	−0.03	(0.08)	−0.03	−0.02	(0.08)		−0.02	−0.02	(0.08)	−0.02
Sleep duration	0.0003	(0.09)	0.0003	−0.03	(0.09)	−0.03	−0.01	(0.09)		−0.005	−0.002	(0.09)	−0.002
Inflammatory marker†	0.12	(0.08)	0.13	0.18	(0.08)	0.19^*^	0.01	(0.08)		0.01	0.07	(0.07)	0.08
Adjusted R^2^	0.18			0.20			0.16				0.17		
F for change in R^2^	1.20			2.77			0.01				0.45		
**BLOCK 3**														
Age (years)	−0.22	(0.09)	−0.24^*^	−0.27	(0.08)	−0.29^***^	−0.29	(0.08)		−0.32^***^	−0.28	(0.08)	−0.30^***^
Education (years)	0.12	(0.08)	0.13	0.12	(0.07)	0.13	0.14	(0.08)		0.15	0.14	(0.08)	0.14
Race	0.22	(0.11)	0.16^*^	0.21	(0.11)	0.16	0.26	(0.11)		0.19^*^	0.24	(0.11)	0.17^*^
GDS Score	−0.11	(0.09)	−0.11	−0.16	(0.09)	−0.16	−0.12	(0.09)		−0.12	−0.13	(0.09)	−0.12
GPM Score	−0.005	(0.08)	−0.005	−0.003	(0.08)	−0.003	−0.01	(0.08)		−0.01	−0.01	(0.08)	−0.02
CIRS Total Score	−0.17	(0.08)	−0.19^*^	−0.14	(0.08)	−0.15	−0.12	(0.08)		−0.13	−0.15	(0.08)	−0.16
BMI	−0.03	(0.08)	−0.03	−0.06	(0.08)	−0.06	−0.02	(0.08)		−0.02	−0.03	(0.08)	−0.03
Sleep Duration	0.01	(0.09)	0.01	−0.03	(0.09)	−0.02	−0.01	(0.09)		−0.005	−0.01	(0.09)	−0.01
Inflammatory Marker†	0.10	(0.08)	0.11	0.20	(0.08)	0.21^**^	0.01	(0.08)		0.01	0.06	(0.08)	0.06
Sleep^X^Inflammation	−0.21	(0.09)	−0.19^*^	−0.20	(0.09)	−0.18^*^	−0.06	(0.10)		−0.05	−0.11	(0.09)	−0.09
Adjusted R^2^	0.21			0.22			0.16				0.17		
F for Change in R^2^	5.30^*^			5.00^*^			0.35				1.28		

Race dichotomously coded as non-Hispanic white = 1, all other = 0; GDS, Geriatric Depression Scale, modified 5-item version; GPM, Geriatric Pain Measure; CIRS, Cumulative Illness Rating Scale; BMI, body mass index; CRP, C-reactive protein; sICAM, soluble Intercellular Adhesion Molecule; TNFα, Tumor Necrosis Factor-alpha; IL6, Interleukin-6. †CRP, sICAM, TNFα, or IL6.

* p < 0.05;

** p < 0.01;

*** *p < 0.001*.

**Figure 1:**
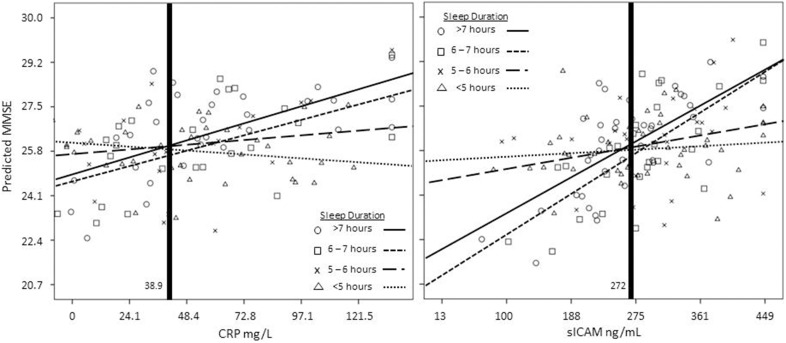
**Interaction between CRP (left panel, mg/L) and sICAM (right panel, ng/mL) and sleep duration**. CRP, sICAM, and MMSE variables were transformed using the Blom transformation prior to analyses. All variables were transformed back to their original metric prior to graphing. Values to the left of the vertical bold lines fall within the regions of significance.

To further explicate the moderating effect in the relationship between inflammation and general cognitive functioning by sleep duration, we graphed the inflammation-global cognitive functioning relationship for older men separately by sleep duration groups (Figure 1; CRP model on the left, sICAM model on the right). As is illustrated, for CRP and sICAM, short sleep duration attenuates the relationship between inflammation and global cognitive functioning. Simple slope analysis revealed that men with short sleep durations (i.e., less than 5 h per night) do not demonstrate a significant relationship between inflammation and global cognitive functioning [*t*_(130)_ = −0.84, *p* > 0.05; *t*_(130)_ = 0.03, *p* > 0.05, respectively for CRP and sICAM]. However, older men with sleep durations greater than 6 h per night show significant positive relationships between inflammation and global cognitive functioning. The highest levels of global cognitive functioning was seen among older men with higher levels of inflammatory markers who slept more than 7 h per night [*t*_(130)_ = 2.82, *p* < 0.01; *t*_(130)_ = 3.13, *p* < 0.01, respectively CRP and sICAM]. The region of significance analysis indicated that differences in global cognitive functioning between sleep duration groups occurred below Blom-transformed values of CRP = −0.025 and sICAM = 0.024 (equivalent to 38.9 mg/L and 272 ng/mL, respectively).

## Discussion

The current study examined the relationships among sleep duration, inflammatory factors, and global cognitive functioning in a sample of older men who are at an increased risk for cognitive difficulties, those who were recovering from acute health changes in an inpatient setting. Our hypothesis that shorter sleep duration would be associated with worse global cognitive functioning was not supported. Similarly, our hypothesis that levels of inflammatory markers would be related to global cognitive functioning was only supported in the sICAM model, and not in models examining CRP, TNFα, or IL6. Our last hypothesis regarding moderation of the inflammation-global cognitive functioning relationship by sleep duration was partially supported. We found that hospitalized older men with short sleep durations do not show any significant relationship between inflammation and global cognitive functioning, while hospitalized older men with sleep durations greater than 6 h per night show significant positive relationships between inflammation and global cognitive functioning.

In community-dwelling older adults, sleep duration has shown inconsistent relationships with cognitive functioning. Our finding in hospitalized older men aligns with investigators who have reported no significant association between sleep duration and cognitive functioning (Nebes et al., 2009). Interestingly, there have been reported negative relationships between abnormally long sleep duration (≥11 h) and general cognitive functioning in older samples (Faubel et al., 2009). Perhaps it is abnormally long sleep durations that are associated with deleterious cognitive changes in hospitalized older men. Our categorization of sleep duration was limited by the scoring protocol of the PSQI. As such, we were not able to examine long sleepers as a separate group, as any abnormally long sleepers would have been lumped into the greater than 7 h per night of sleep group. Future research should continue to explore these relationships. Another potential explanation for the lack of associations between sleep duration and global cognitive functioning may be the underlying poor health status of our sample. Physical health is a robust predictor of late-life cognitive functioning (Zelinski et al., 1998), and late-life sleep disturbances are largely related to health status (Grandner et al., 2012). Our sample may have been too homogenous regarding health status as we controlled for depression, pain, comorbidity severity/burden, and BMI in a sample of older men all recovering from an acute health event.

An innovation of our study was the examination of markers of inflammation and cognitive functioning in the context of sleep duration. Increased levels of inflammatory markers are associated with a number of negative health outcomes in older people. Previous studies have observed negative relationships between inflammatory markers and cognitive functioning in late-life (Gunstad et al., 2006; Baune et al., 2008), although these studies primarily focused on healthy older adults without consideration of sleep. Interestingly, it has been suggested that a potential route through which higher circulating levels of cytokines and their downstream products may negatively impact cognitive functioning is through “impaired sleep regulation” (Wilson et al., 2002). Elevations of inflammatory markers in response to acute health events is a normal innate immune response (Heinrich et al., 1990; Dantzer and Kelley, 2007). Perhaps individuals who had sufficient immune system capacity to respond to physiological insult with elevated inflammatory levels were also healthier patients, or at least, had greater physical and mental reserves upon which to draw. Adequate sleep duration may facilitate immune functioning and recovery. Such an interpretation is consistent with the moderation results which indicated that older men who slept more than 6 h per night demonstrated a positive relationship between inflammatory markers and global cognitive functioning. In the case of CRP, an acute phase protein produced by the liver, this might reflect better hepatic function. For sICAM-1, which is essential to the migration of leukocytes to sites of inflammation, elevated levels during acute health challenges might reflect appropriate recruitment and migration of leukocytes via vascular endothelial cells. Short sleep durations may dampen these processes, while normal sleep durations may promote or enable these processes.

The present study has several limitations. Our findings pertain to hospitalized older men. While hospitalized older men are at increased risk for future decline (Martin et al., 2011; Wilson et al., 2012), and as such represent an important group to be studied, results may not generalize to non-hospitalized older adults or to older women. Also, cognitive functioning was assessed with a single global indicator (MMSE). Future studies should collect multiple indicators of sleep duration (e.g., self-reported prospective and retrospective, actigraphy, polysomnography), along with examination of the relationship among various domains of cognition, such as executive functioning, attention, and working memory. In our sample, both CRP and IL-6 levels were generally high and well above reported national averages for non-hospitalized individuals (Ford, 1999). As the current sample was drawn from an inpatient rehabilitation unit, we expected elevations in inflammatory markers. To be conservative, we trimmed extreme outliers at the highest 5% of values and normalized the data through Blom transformations (Blom, 1958). Additionally, it was not possible to exclude individuals with inflammatory diseases on admission or to ascertain whether we completed blood draws while patients were in the acute phase response of their illness, which is considered a normal response to bodily insult and include significant increases in both IL-6 and CRP (Heinrich et al., 1990). In fact, the region of significance analysis (see Figure 1) suggested that differences in global cognitive functioning between sleep duration groups occurred in the lower half of the range of the inflammation distributions. Future investigations might employ more sophisticated analytical techniques which could more clearly parse out the unique contributions of sleep and inflammation on late-life cognitive functioning. Lastly, there are many medications with known inflammatory consequences, and these were not accounted for in the present analysis.

We discovered that the relationship between inflammatory markers and global cognitive functioning was dependent on sleep duration in a group of older men hospitalized for rehabilitation services. These relationships were observed after controlling for age, education, race, comorbidity severity/burden, depression, pain, and BMI. Future studies of cognitive functioning in hospitalized older adults would be wise to include measures of self-reported sleep duration and determinations of circulating levels of CRP and sICAM. Additionally, as sleep remains malleable well into late-life (Dzierzewski et al., 2010), future work should explore sleep interventions as a means promote recovery and/or delay cognitive decline following hospitalizations. Lastly, the results of the present study highlight the importance of context when investigating predictors, especially inflammation, of late-life cognitive functioning.

## Author contributions

All authors contributed substantially with the conception and study design, data acquisition and analysis, data interpretation, manuscript drafting and revisions, and manuscript approval.

### Conflict of interest statement

The authors declare that the research was conducted in the absence of any commercial or financial relationships that could be construed as a potential conflict of interest.
